# Selection of Potential Probiotic Yeasts from Dry-Cured Xuanwei Ham and Identification of Yeast-Derived Antioxidant Peptides

**DOI:** 10.3390/antiox11101970

**Published:** 2022-09-30

**Authors:** Jiaming Cai, Lujuan Xing, Wangang Zhang, Lijuan Fu, Jian Zhang

**Affiliations:** Key Laboratory of Meat Processing and Quality Control, MOE, College of Food Science and Technology, Nanjing Agricultural University, Nanjing 210095, China

**Keywords:** antioxidant, probiotic, *Yamadazyma triangularis*, yeast-derived peptides, HPLC–MS/MS

## Abstract

The aim of this study was to select potential probiotic yeasts from dry-cured Xuanwei ham and investigate yeast-derived antioxidant peptides. The results showed that two strains (XHY69 and XHY79) were selected as potential probiotic yeasts and identified as *Yamadazyma triangularis*. The two yeasts showed tolerance under pH 2.5 and 1% bile salt, in addition to protease activity, auto-aggregation, antibacterial, and antioxidant activities. The peptide fraction (MW < 3 kDa) isolated from XHY69 fermentation broth, named XHY69AP, showed higher radical scavenging activities than glutathione at a concentration of 4.5 mg/mL (*p* < 0.05). The fraction (AP-D10) was purified from XHY69AP by gel filtration chromatography and reversed-phase high performance liquid chromatography, and then further identified by a UHPLC-LTQ-Orbitrap mass spectrometer. The molecular weight of all 55 purified sequences was distributed between 0.370 and 0.735 kDa. Among these seven novel peptides, Tyr-Pro-Leu-Pro (YPLP), Ala-Gly-Pro-Leu (AGPL), Gly-Pro-Phe-Pro (GPFP), and Ala-Pro-Gly-Gly-Phe (APGGF) were identified. All sequences were abundant in hydrophobic amino acids, especially proline residue. Among these novel peptides, YPLP possessed the highest ABTS scavenging rate (75.48%). The present work selects two new probiotic potential yeasts from dry-cured Xuanwei ham that are effective to yield novel antioxidant peptides.

## 1. Introduction

Xuanwei ham, a traditional Chinese dry-cured meat product, presents a unique flavor, color, and texture due to its natural maturation process. More importantly, suitable temperature and humidity conditions during the maturation of ham provide an excellent environment for the growth of beneficial yeast [1]. The predominant yeasts reported in Spain Iberian ham were *Debaryomyces hansenii*, *Pichia* spp., and *Candida* spp. [2]. In recent years, the focus on yeast has gradually shifted from fermentation starter cultures to probiotic strains.

Probiotics are defined as “live microorganisms, which when administered in adequate amounts, confer a health benefit on the host” (FAO/WHO, 2001) [3]. Probiotic yeast is beneficial to the host’s health through colonization, competition for intestinal adhesion, and balance of the intestinal microbiota [4], with *Saccharomyces cerevisiae* being the most commonly recognized probiotic yeast [5]. Previously, fermented foods and drinks have been reported to possess potential probiotic yeasts, including *Candida*, *Pichia*, *Torulaspora*, and *Metschnikowia genera* [6,7]. Moreover, Klemashevich, et al. [8] reported that the major contribution of probiotics to health was not only related to the live microorganisms, but also the metabolites of microorganisms. Postbiotics are functional bioactive compounds obtained by microbial fermentation and include proteins, carbohydrates, lipids, vitamins, organic acids, or other complex molecules [9]. Postbiotics can have direct and indirect effects on the intestinal microbiota [10], anti-inflammatory responses [11] and host immune function [12]. The metabolite of probiotic yeast could be considered as a candidate for postbiotic elements and offer a positive effect on the host [13]. Therefore, it is essential to explore novel probiotics and their main metabolites, while there are no published studies about the probiotic yeast isolated from dry-cured Xuanwei ham.

Overproduction of reactive oxygen species (ROS) could induce oxidative stress, destroy the biomacromolecule, and therefore cause human diseases, such as cardiovascular disease, inflammatory disease, and diabetes [14]. Antioxidant peptides are important products to protect the body from harm caused by ROS, which are expected to be used as functional nutrients for humans [15]. Generally, bioactive peptides can be divided into natural and synthetic peptides, while synthetic peptides are expensive, low-yielding, and environmentally unfriendly. Interestingly, microorganism-derived peptides, one kind of the natural peptides, have the advantages of being environmentally friendly, low cost, readily accessible, along with potential for large-scale fermentation production.

Yeast is considered as an effective source for producing antioxidant peptides. In recent years, the yeast-derived antioxidant peptide is a research highlight. Vieira, et al. [16] selected peptides (MW < 5 kDa) from the brewer’s spent yeast autolysates that decreased the ROS level of Caco-2 cells. Mirzaei, et al. [17] obtained the peptide (VCK-9) with nine amino acids from *Kluyveromyces marxianus,* which showed high DPPH and ABTS scavenging capacity. Currently, yeast-derived peptides can be divided into the following three categories: bioactive peptides released by yeast cells; peptides from yeast fermentation products; and yeast extracts produced by autolysis or hydrolysis of yeast cells [18]. However, limited studies have focused on the antioxidant peptides derived from probiotic yeast fermentation broth.

Thus, the purpose of this work was to select potential probiotic yeasts from dry-cured Xuanwei hams according to their probiotic properties and obtain antioxidation peptides from yeast by using ultrafiltration, gel filtration chromatography, reversed-phase HPLC, and a UHPLC-LTQ-Orbitrap mass spectrometer. The present study will provide a comprehensive exploration of new probiotic yeast resources and yeast-derived novel antioxidant peptides.

## 2. Materials and Methods

### 2.1. Chemicals and Materials 

The 1,1-diphenyl-2-picrylhydrazyl (DPPH) and 2,2′-azino-bis(3-ethylbenzothiazoline-6-sulfonic acid) (ABTS) were purchased from Sigma Chemical Co. (St. Louis, MO, USA). The glutathione (GSH) and other reagents were purchased from Beijing Solarbio Science & Technology Co., Ltd. (Beijing, China). Peptide sequence (98% purity) was synthesized in Nanjing Jinsirui Science & Technology Biology Corp (Nanjing, China).

### 2.2. Ham Sampling and Yeast Isolation

Xuanwei ham was fermented for 24 months (Laopujia Ham Co., Ltd., Yunnan, China) and 3 hams were randomly selected for sampling. The surface of Xuanwei ham was wiped with 75% ethanol, and 25 g of biceps femoris muscle at 2 cm under the surface of the ham was cut up and placed into 225 mL sterile normal saline. Yeast was selected in rose Bengal medium, forming pink, round and convex colonies. The colony was purified in YPD medium by the streak plate method. The 108 isolates were tested for subsequent analyses.

### 2.3. Determination of Probiotic Properties

#### 2.3.1. Growth at pH 2.5 and in Bile Salts

The growth abilities of all isolates were tested at pH 2.5 and bile salts using a methodology adapted from the work of Zoumpopoulou, et al. [19]. Firstly, the 108 isolates were incubated in YPD broth for 24 h at 28 °C. YPD broth was acidified with 2 M HCl to reach pH 2.5 and added to 1% (*w*/*v*) bile salts, respectively. Then, 10^6^ cells/mL of the strain were incubated in modified YPD broth for 48 h at 28 °C. The strain cultured in the YPD broth without bile salts and acidification was set as the control.

#### 2.3.2. Proteinase Activity

Briefly, the isolates were inoculated in skimmed milk powder medium (YPD medium with 10% skimmed milk powder) for 3 d at 28 °C. The proteinase activity of yeasts was shown by the size of the hydrolysis circle. 

#### 2.3.3. Auto-Aggregation Capacity

The 3 mL of yeast fermentation solutions was centrifuged (12,000× *g*, 5 min) to collect strain cells. The cells were washed twice with sodium chloride solution (0.9%, *m*/*v*) and resuspended in PBS buffer (3 mL), with incubation at 37 °C. Cell suspensions were selected after 0 h, 2 h, 4 h, and 24 h of incubation and measured at 517 nm, respectively. The auto-aggregation was tested by the following equation:

$$\text{Auto-aggregation}\;(\%)=\left[1-\left(\frac{A_{t}}{A_{0}}\right)\right]\times \;100$$

A_0_ and A_t_ are the absorbance of cell suspensions before and after incubation.

#### 2.3.4. Antimicrobial Activity

The pathogen indicator bacteria, *Escherichia coli*, *Staphylococcus aureus,* and *salmonella* sp., were coated on LB solid medium. To measure the antimicrobial activity of yeast to pathogen bacteria, 200 μL yeast cultures were added to the indicator bacteria LB medium. The antimicrobial capacity of yeasts was measured by the size of the inhibition zone.

#### 2.3.5. Antioxidant Activity of Yeast

Yeasts were fermented in YPD broth for 3 d at 28 °C and the total antioxidant capacity was measured using the T-AOC method kit (Jiancheng Chemical Regent Co., Nanjing, China).

### 2.4. Yeast Identification

Yeast DNA was extracted using the fungus DNA Extraction Kit (Solarbio, Beijing, China) and the purification of DNA was performed by a micro-spectrophotometer (NanoDrop, Thermo Fisher Scientific, Waltham, MA, USA). The ITS1-5.8S rRNA-ITS2 region was amplified by PCR using the primer pair ITS1 and ITS4 to identify the gene sequences. Yeast was identified by the alignment of sequences to similar fungal genes in NCBI and by comparison of the phylogenetic tree.

### 2.5. Peptide Content

The content of peptides in supernatants was detected by OPA solution based on the method of Xing, et al. [20]. Casein, ranging in concentration from 0.1 to 1.0 mg/mL, was set as the standard curve to measure the peptide content.

### 2.6. Determination of Antioxidant Activity 

#### 2.6.1. DPPH Radical Scavenging Activity

A mixture of 800 μL sample and 1 mL DPPH solution (0.2 mM) was reacted in the dark for 30 min and then the absorbance value was detected at 536 nm (A_sample_). The blank group was ethanol mixed with deionized water (A_blank_). The control group was DPPH solution mixed with deionized water (A_control_). The activity was assayed using the following equation:

$$\mathrm{DPPH}\;\mathrm{radical}\;\mathrm{scavenging}\;(\%)=\left[1-\left(\frac{A_{\mathrm{sample}}-A_{\mathrm{blank}}}{A_{\mathrm{control}}-A_{\mathrm{blank}}}\right)\right]\;\times \;100$$

#### 2.6.2. Hydroxyl Radical Scavenging Activity

A mixture of 0.6 mL of the sample, 0.6 mL of 1,10-phenanthroline solution (5 mM), 0.6 mL of FeSO_4_ solution (5 mM), and 0.4 mL of PBS buffer was added to 0.8 mL of H_2_O_2_ (0.1%) and incubated at 37 °C for 1 h. The absorbance of mixture was detected at 536 nm (A_sample_). The sample was replaced by ultrapure water as the damage group (A_damage_). The sample and H_2_O_2_ were replaced by ultrapure water as the undamaged group (A_undamage_). The activity was tested by the equation as follows:

$$\mathrm{Hydroxyl}\;\mathrm{radical}\;\mathrm{scavenging}\;(\%)=\frac{A_{\mathrm{sample}}-A_{\mathrm{damage}}}{A_{\mathrm{undamge}}-A_{\mathrm{damage}}}\;\times \;100$$

#### 2.6.3. ABTS Radical-Scavenging Activity

A mixed solution of 0.2 mL ABTS (7.4 mM) and 0.2 mL K_2_S_2_O_8_ (2.6 mM) reacted overnight and then was diluted with ethanol, until the OD_734_ value reached around 0.70. Samples (0.2 mL) were added to ABTS solution (0.8 mL), reacted for 6 min and then the absorbance was read at 734 nm (A_sample_). The sample was replaced by ethanol as the control group (A_control_). The activity was tested using the following equation:

$$\mathrm{ABTS}\;\mathrm{radical}\;\mathrm{scavenging}\;(\%)=\frac{A_{\mathrm{control}}-A_{\mathrm{sample}}}{A_{\mathrm{control}}}\;\times \;100$$

#### 2.6.4. Reducing Power

Reducing power was assessed as described by Ge, et al. [21]. L-cysteine, ranging in the concentration from 0.1 to 2.0 mM, was used for the standard curve. The reducing power of peptides and GSH was expressed as μmol/L L-cysteine.

### 2.7. Separation and Identification of Antioxidant Peptides 

#### 2.7.1. Peptide Isolation and Purification by Ultrafiltration (UF)

Yeasts were fermented in YPD broth for 3 d at 28 °C. The fermentation broth was centrifuged (12,000× *g*) at 4 °C for 10 min and then filtered using the 0.22-μm membrane. The supernatant was separated through a 3 kDa ultrafiltration membrane (Millipore, Bedford, MA, USA). All fractions were collected and measured.

#### 2.7.2. Gel Filtration Chromatography (GFC)

The fraction of 20 mg/mL was separated by a Sephadex-G10 column (160 × 70 cm) using an AKTA purifier system (Amersham Pharmacia Biotech, Amersham, UK). The sample was eluted with ultrapure water at 1.5 mL/min and monitored at 214 nm. 

#### 2.7.3. Preparative Reversed-Phase High-Performance Liquid Chromatography (RP-HPLC)

The fraction was separated by RP-HPLC (Waters Inc., Milford, MA, USA) using a C18 column (1.7 μm, 2.1 × 150 mm, Waters) at a flow rate of 2.5 mL/min. A non-linear gradient of solvent A (distilled water with 0.1% formic acid) to solvent B (0.1% formic acid in acetonitrile) was monitored at 214 nm. All fractions were collected and freeze-dried for further analysis.

#### 2.7.4. Peptide Identification by LC-MS/MS

The fraction sequences were analyzed using a UHPLC-LTQ-Orbitrap mass spectrometer with a C18 column (5 µm, 250 mm × 3 mm). The filtrate was re-dissolved at 0.5 mg/mL with ultrapure water. The linear gradient with solvent A (0.1% formic acid) and solvent B (100% CAN) was operated as follows: 0–2 min, 3–3% B; 2–17 min, 3–40% B; 17–18 min, 40–95% B; 18–28 min, 95–95% B; 28–28.5 min, 95–2% B, 28.5–33 min, 2–2% B. Raw data were visualized and analyzed by PEAKS (Version Xpro, Waterloo, ON, Canada) software to obtain peptide sequences.

### 2.8. Statistical Analysis

The major tools and databases for analyzing functional peptides were UniProt (www.Uniprot.org, accessed on 15 January 2022), BIOPEP (http://www.uwm.edu.pl/biochemia, accessed on 15 January 2022), and the Pepdraw tool (http://pepdraw.com/, accessed on 15 January 2022).

All data were expressed as mean ± standard error and statistically analyzed by SPSS 20.0 software (SPSS, Chicago, IL, USA). The significant differences (*p* < 0.05) were evaluated by Duncan’s multiple-range test in one-way analysis of variance and Student’s *t*-test.

## 3. Results and Discussion

### 3.1. Probiotic Potential of Yeast

#### 3.1.1. Ability to Grow at pH 2.5 and in Bile Salts

Probiotics are resistant to the intestinal environment with gastric acid and bile salts [22]. To select the yeast strains with the property of growing at pH 2.5 and high bile salts, 108 isolates were tested (Table 1). The results showed that 41 isolates could grow at pH 2.5 and 50 isolates could grow at 1% bile salt. A total of 27 isolates could grow both at pH 2.5 and in 1% bile salt. In the present study, almost 50% of the isolates can tolerate bile salt, probably because they were originated from dry-cured Xuanwei ham, which has a high salt content (around 8%) in the final product. Among them, XHY23, XHY28, and XHY50 exhibited excellent growth abilities. In addition, yeast probiotics showed a higher resistance to the intestinal environment compared to bacterial probiotics [23].

#### 3.1.2. The Proteinase Activity of Yeasts

Protease activity as a reference indicator of probiotics reflects protein degradation capacity. As shown in Table 1, more than half of the isolates presented protease activity and 11 strains with higher protease activities had the potential to degrade the large molecules of protein into bioactive peptides. In addition, strains with higher protease activity implied promising fermentation characteristics and contributed to the production of bioactive components [24]. Combining all indicators in Table 1, 27 strains that could grow in the simulated intestinal environment with a higher protease activity were selected for the following parts.

#### 3.1.3. The Auto-Aggregation Ability of Yeasts

The high auto-aggregation of strains promoted adhesion to host epithelial cells and increased persistence in the gastrointestinal tract [25]. In addition, the colonization of the gut by probiotics created a defense system to prevent the invasion of pathogens [26]. The auto-aggregation abilities of isolates incubated for 0 h, 2 h, 4 h, and 24 h are shown in Table 2. The auto-aggregation values gradually increased with extended incubation time and reached the maximum at 24 h of incubation. It is worth noting that 13 strains showed outstanding auto-aggregation capacity (more than 90% at 24 h), among which XHY21 exhibited the highest auto-aggregation capacity (95.44%). High auto-aggregation yeasts could prevent the invasion of other pathogenic microorganisms through the formation of biofilms in vivo [4]. Consequently, the 13 strains with high auto-aggregation had the potential to prevent pathogenic microorganisms from disrupting the intestinal balance.

#### 3.1.4. The Antibacterial Properties of Yeasts

The antibacterial activity of yeasts was determined against *Escherichia coli*, *Staphylococcus aureus*, and *Salmonella* sp. (Table 2). The results showed that most isolates displayed obvious antibacterial activity on *Salmonella* sp. and half of the isolates were effective against *Staphylococcus aureus*. The main reason for the antimicrobial activity of probiotics is the production of antibacterial components, such as bioactive peptides [14], organic acids, and hydrogen peroxide [27]. However, fewer isolates presented inhibition capacity against *Escherichia coli*, which is consistent with the result of Binetti, et al. [28], who found that yeast strains isolated from cheese presented a poor inhibitory capacity against *Escherichia coli*.

#### 3.1.5. The Antioxidant Activity of Yeasts

**Figure 1:**
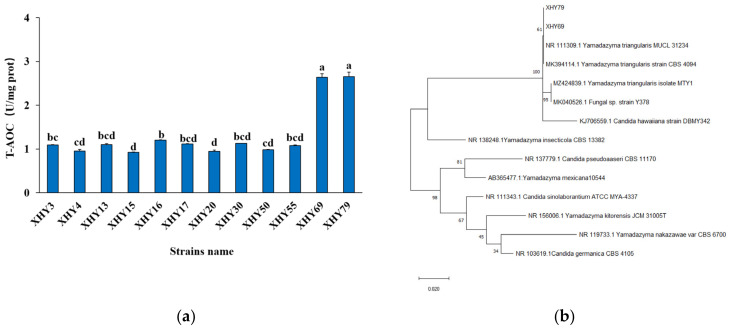
Determination of potential probiotic yeasts. (**a**) The total antioxidant capacity of 12 strains. (**b**) Phylogenetic tree of amplified sequences of ITS rRNA gene of XHY69 and XHY79 with similar fungal genes retrieved from NCBI. Different letters (a–d) in the T-AOC index represent significant differences (*p* < 0.05, *n* = 6).

As shown in Table 2, the DPPH scavenging values of 27 isolates were all above 74%, among which 12 isolates were over 84%. Combining the results of DPPH radical scavenging activity, antibacterial properties, and auto-aggregation ability, twelve strains were selected for the measurement of total antioxidant capacity (T-AOC). Two strains (XHY69 and XHY79) showed significantly higher antioxidant activity than the other strains among the twelve yeasts (Figure 1a), with T-AOC being over 2.5 U/mg prot (*p* < 0.05). This phenomenon could be attributed to the fact that XHY69 and XHY79 produced more metabolites with antioxidant activity. Therefore, XHY69 and XHY79 strains were considered as potential probiotics for further strain identification.

The antioxidant capacities of XHY69 and XHY79 were remarkably stronger than the yeast strains reported by Goktas, et al. [29] and bacteria strains reported by Amaretti, et al. [30]. The antioxidant mechanisms of most probiotics in vivo have been summarized as those that produce antioxidant metabolites, up-regulate antioxidase activities, chelate metal ions, and regulate related signaling pathways [31].

#### 3.1.6. Strain Identification

In order to characterize strains at the species-level, highly conserved ITS rRNA gene sequences of XHY69 and XHY79 were amplified by PCR and were matched by using BLAST. The sequences with similarities larger than 98% were compared in a phylogenetic tree. As shown in Figure 1b, XHY69 and XHY79 were identified as *Yamadazyma triangularis,* which was also identified in Spain Iberian ham and Danish cheese [32]. *Yamadazyma triangularis* from Danish cheese was applied in cheese production and found to enhance the amino acid utilization and aroma compounds of products [33]. However, the potential probiotic and antioxidant properties of *Yamadazyma triangularis* have not been reported.

### 3.2. Purification and Analysis of Yeast-Derived Peptides

#### 3.2.1. Selection of Yeast-Derived Peptides by UF

The extracellular metabolites of microorganisms are complex, including polypeptides, polysaccharides, organic acids, and nucleotides. The extracellular peptides derived from yeast are mainly released from yeast cells, degraded by proteases during fermentation, and produced by the autolysis of yeast cells [18]. The metabolites of probiotics generally possess a highly bioactive capacity and peptides derived from probiotics play a vital part in antioxidant properties [31].

**Figure 2:**
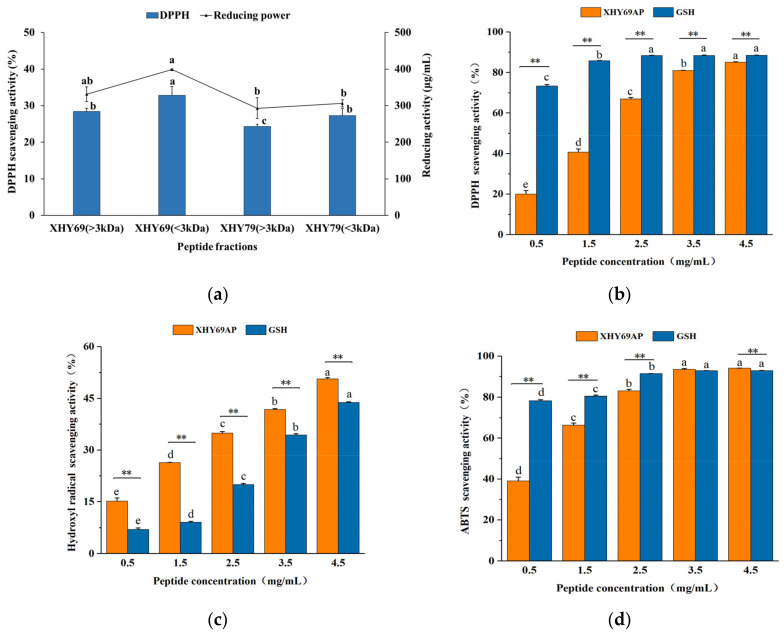
Separation of the antioxidant peptides. (**a**) The antioxidant activities of different peptide fractions separated by ultrafiltration at a concentration of 1 mg/mL. (**b**–**d**) The antioxidant activities of XHY69AP and GSH at different concentrations. Different letters in the same index on different peptide fractions are significantly different (*p* < 0.05, *n* = 6). Different letters in the same peptide indicate significant differences (*p* < 0.05, *n* = 6). ** p < 0.01 indicates a significant difference between XHY69 and GSH at the same concentration.

Owing to their strong antioxidant properties, the XHY69 and XHY79 derived peptides were explored in the current study. The DPPH scavenging and reducing power of the peptides were affected by different strains and molecular weight (Figure 2a). The XHY69 strain had higher antioxidant activities than the XHY79 strain (*p* < 0.05). In addition, for the same strain, the small peptides under 3 kDa had better antioxidant ability compared with large molecular peptides. A similar report also confirmed that peptide fractions (MW < 3 kDa) from *Kluyveromyces marxianus* [34] had higher antioxidation capacity than large molecular peptides, mainly because small molecular peptides have smaller spatial structures and short-chain active centers [35], which present a stronger free radical scavenging effect and interfere with the oxidation process.

In order to select a highly concentrated peptide fraction with a strong antioxidant capacity, the content of peptides was also determined (Table S1 in Supplementary Materials). The peptide content of all fractions was more than 87%. The XHY69 (<3 kDa) presented the highest peptide concentration of 97.69%, and thus was selected for further research, which was named XHY69AP.

#### 3.2.2. The Antioxidant Activity of XHY69AP

The antioxidant capacity of XHY69AP was assessed by determining DPPH, hydroxyl, and ABTS scavenging activity using GSH as the control. The scavenging rates of XHY69AP and GSH increased with the elevation of their concentration (Figure 2b–d). The scavenging rate on DPPH of XHY69AP (85.09%) was lower than that of GSH (88.49%) at 4.5 mg/mL concentration, while the scavenging rate on ABTS of XHY69AP (94.15%) was higher than that of GSH (92.96%, *p* < 0.01). Meanwhile, XHY69AP presented a stronger hydroxyl radical scavenging rate than GSH at the same concentration (*p* < 0.01). These results indicate that XHY69AP possessed high antioxidant activity, thus, XHY69AP was applied for further purification to obtain specific antioxidant peptides.

#### 3.2.3. Separation of XHY69AP by GFC

**Figure 3:**
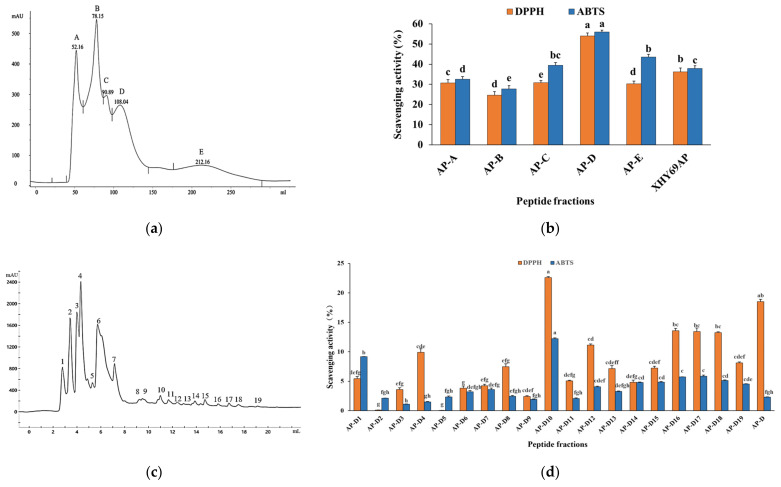
Purification of the antioxidant peptides. (**a**) Sephadex G-10 chromatogram of XHY69AP. (**b**) DPPH and ABTS radical-scavenging activity of purified peptide fractions at a concentration of 1 mg/mL. (**c**) RP-HPLC chromatogram of AP-D10 fraction. (**d**) DPPH and ABTS radical-scavenging activity of purified peptide fractions at a concentration of 0.2 mg/mL. Different letters in the same index indicate significant differences (*p* < 0.05, *n* = 6).

The XHY69AP was fractionated using a size exclusion chromatography column (G-10). Five fractions were obtained from XHY69AP and selected according to their DPPH and ABTS radical-scavenging capacity, as shown in Figure 3a,b. The DPPH and ABTS scavenging rates were highest in fraction AP-D (52.05% and 55.98%), followed by fractions AP-E, AP-C, AP-A, and AP-B (*p* < 0.05).

In general, the smaller molecular weight components were later separated in gel filtration chromatography. These results suggest that the fraction with a low molecular weight (AP-D and AP-E) of XHY69AP had higher antioxidant activity. Previous research has indicated that the antioxidant capacity of active peptides was affected by their molecular weight distribution [36] and small peptides possessed a strong antioxidant effect. Similarly, the fraction with a smaller molecular weight from *Saccharomyces cerevisiae* obtained a better radical-scavenging rate than larger fractions [37]. Considering AP-D had the highest antioxidant capacity, it was used for the following purification.

#### 3.2.4. Purification of AP-D by Reverse Phase-HPLC

RP-HPLC is a commonly used method for the purification of small molecule peptides and is suitable for the separation of non-polar, polar, or ionic compounds. AP-D was fractionated by RP-HPLC, relying on hydrophobic interactions to obtain higher activity fractions. A total of 19 different fractions were separated from AP-D (Figure 3c). As shown in Figure 3d, AP-D10 presented the highest DPPH and ABTS scavenging rate of all the isolated fractions (*p* < 0.05). The late peak time of AP-D10 fraction indicates that it had a high degree of hydrophobicity. Hence, the sequence of AP-D10 was further characterized.

#### 3.2.5. Identification of AP-D10 by LC-MS/MS

**Figure 4:**
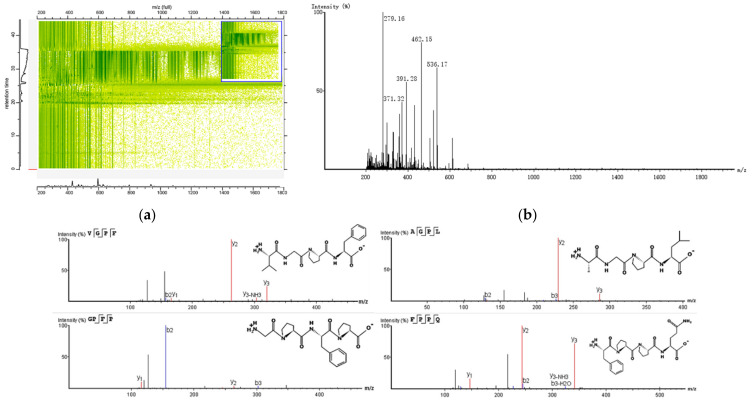

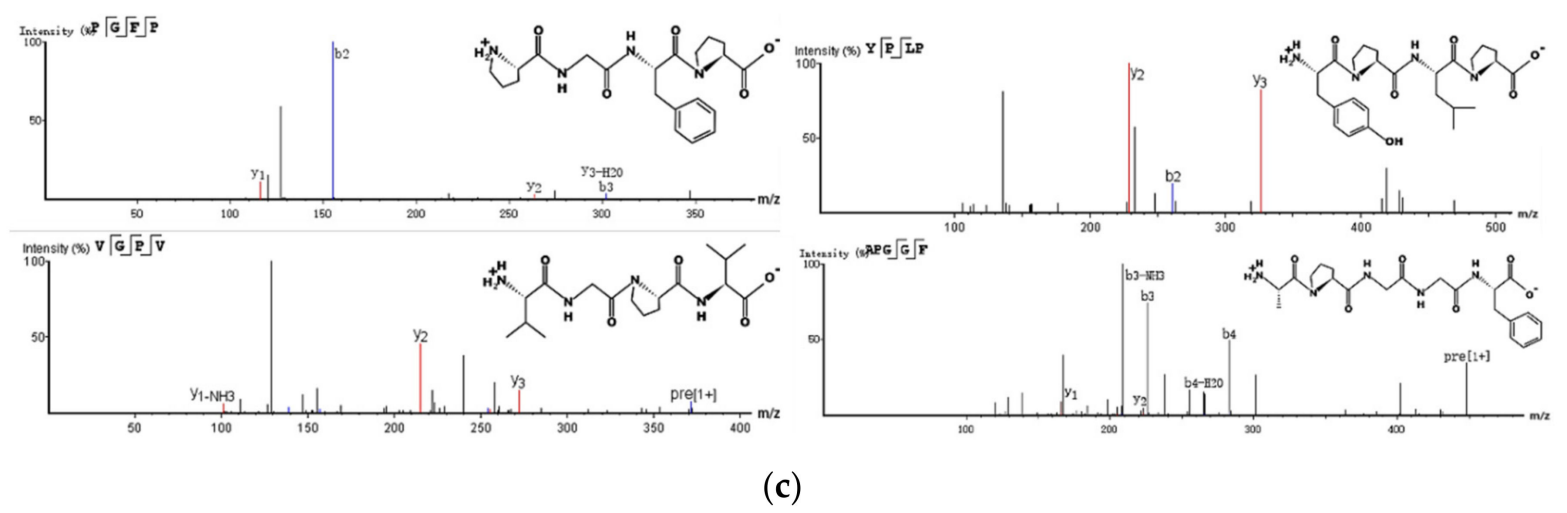
Identification of the molecular mass and amino acid sequence of the AP-D10 peptide using the UHPLC-LTQ-Orbitrap mass spectrometer. (**a**) Total ion chromatography of AP-D10. The ionic intensity is shown in green. (**b**) Mass spectrum of AP-D10. (**c**) Identification of amino acid sequence by MS/MS spectrum and the structure of the sequences.

The peptide sequence was determined by LC-MS/MS and the physicochemical properties were evaluated by the Pepdraw tool. As shown in Figure 4a,b, the mass spectrometry peaks were mainly concentrated in the 200–600 *m*/*z* range, with a peak time of 20–35 min. A total of 55 peptides were detected in AP-D10, with the molecular weight ranging from 0.37 to 0.735 kDa (Table S2 in Supplementary Materials).

To ensure high confidence in the sequences, the threshold of the ALC value was set at 95%. As shown in Table 3, a total of eight peptide sequences were selected, including Phe-Pro-Pro-Gln (FPPQ), Val-Gly-Pro-Phe (VGPF), Aal-Gly-Pro-Leu (AGPL), Tyr-Pro-Leu-Pro (YPLP), Val-Gly-Pro-Val (VGPV), Gly-Pro-Phe-Pro (GPFP), Pro-Gly-Phe-Pro (PGFP), and Aal-Pro-Gly-Gly-Phe (APGGF). The molecular weight of the peptides was less than 0.5 kDa and the structure of the eight peptides is shown in Figure 4c.

The peptide sequences possessed a large proportion of hydrophobic amino acids, particularly in AGPL and APGGF. The active characteristics of peptides were associated with amino acid sequences and physicochemical properties. Previous studies have shown that the hydrophobic amino acids had a beneficial effect on the antioxidant capacity and the hydrophobicity enabled peptides to react readily with hydrophobic radicals and lipids [38]. The results showed that the three most abundant amino acids were Pro, Phe, and Gly, and eight peptide sequences contained Pro residue. Similar reports indicated that the antioxidant peptides from the cheese fermented by *Lactobacillus helveticus* contained at least two Pro residues [39], and the Pro residue in purified peptides had a positive effect on antioxidant activity. You, et al. [40] also found that Pro, Val and Tyr could enhance the activities of the antioxidant. In addition, the antioxidant properties of peptides were influenced by N- terminal and C-terminal amino acid residues. The Leu residues at the N-terminal and C-terminal of the peptide played important roles in radical scavenging activities [41]. Moreover, the peptide contained aromatic amino acids Trp, Tyr, and Phe at the C-terminal, which contribute to high free radical scavenging [36]. The Val, Pro, and Phe residues were enriched at the N-terminal and C-terminal of AP-D10. This is consistent with the sequences in several antioxidant peptides, which were rich in Gly, Val, Pro, and Leu at the N-terminal region [42]. 

The sequence comparison of AP-D10 from the BIOPEP database and Uniport database showed that seven peptide sequences have not been reported in the previous studies, except for Val-Gly-Pro-Val with enzyme (ACE)-inhibitory activity found by Fu, et al. [43]. The parent proteins of the eight peptides were predicted by UniPort. The peptides were mainly derived from transporter protein, mitochondrion, and protein kinase, which were connected with the autophagic degradation pathway, cell wall synthesis, protein degradation pathways, mitochondrial metabolic pathways, amino acid transportation, and ion transport. 

The eight peptides were synthesized to further verify their antioxidant capacity. In the comparison between the eight peptides (Table 4), YPLP had stronger ABTS radical scavenging activity (75.48%). Thus, YPLP could play the dominant role in the antioxidant capacity of AP-D10. In contrast, other synthesized peptides presented a lower radical scavenging rate. Fu, et al. [44] indicated that the crude peptides from Xuanwei ham had better biological activity than individual peptides, which was probably due to their synergistic effect.

## 4. Conclusions

In this study, it was first shown that *Yamadazyma triangularis* (XHY69 and XHY79) isolated from Xuanwei ham presented potential probiotic properties. The XHY69-derived peptides with high antioxidant activity were purified, with the AP-D10 fraction showing stronger antioxidant capacity. After identification, eight peptide sequences were obtained from AP-D10 and the synthetic peptide YPLP presented the highest ABTS scavenging activity (75.48%). Thus, XHY69 is a promising probiotic strain and the XHY69-derived peptides exhibit strong antioxidant capacity. Further research will focus on revealing the antioxidant mechanisms of XHY69-derived peptides in vivo and the effect of XHY69 on the sensory properties of fermented products.

## Figures and Tables

**Table 1:** Growth of 108 isolates at pH 2.5 and 1% bile salts, and the proteinase activity of isolates.

Yeast Strain Name	Growth at pH 2.5	Growth at 1% Bile Salt	Proteinase Activity	Yeast Strain Name	Growth at pH 2.5	Growth at 1% Bile Salt	Proteinase Activity
XHY1	-	-	-	XHY55	-	++	+
XHY2	++	-	-	XHY56	-	++	-
XHY3	+	++	+	XHY57	-	-	-
XHY4	+	++	+	XHY58	-	++	+
XHY5	++	-	-	XHY59	+	+	+
XHY6	-	-	+	XHY60	-	-	+
XHY7	+	-	+	XHY61	-	-	-
XHY8	++	-	-	XHY62	-	-	+
XHY9	-	++	-	XHY63	-	+	-
XHY10	-	-	-	XHY64	-	-	+
XHY11	+	-	-	XHY65	-	-	-
XHY12	+	-	-	XHY66	-	-	-
XHY13	+	++	+	XHY67	-	-	+
XHY14	-	-	-	XHY68	-	-	+
XHY15	+	++	+	XHY69	+	++	+
XHY16	+	+	+	XHY70	-	++	++
XHY17	++	++	+	XHY71	-	-	-
XHY18	+	++	++	XHY72	+	-	+
XHY19	-	-	-	XHY73	-	-	-
XHY20	+	++	-	XHY74	-	-	+
XHY21	+	++	+	XHY75	-	-	-
XHY22	-	-	--	XHY76	-	+	-
XHY23	++	++	+	XHY77	-	-	-
XHY24	-	-	-	XHY78	-	-	-
XHY25	-	-	-	XHY79	+	++	+
XHY26	-	+	+	XHY80	-	-	+
XHY27	-	+	++	XHY81	-	++	+
XHY28	++	++	+	XHY82	-	-	+
XHY29	-	++	+	XHY83	-	++	++
XHY30	+	+	+	XHY84	-	++	-
XHY31	-	++	-	XHY85	-	-	++
XHY32	-	++	-	XHY86	-	-	-
XHY33	-	-	+	XHY87	-	-	+
XHY34	-	-	-	XHY88	++	-	+
XHY35	+	++	+	XHY89	+	+	+
XHY36	-	+	-	XHY90	++	-	+
XHY37	-	-	-	XHY91	-	-	-
XHY38	-	-	+	XHY92	+	++	++
XHY39	-	+	-	XHY93	+	+	++
XHY40	-	-	-	XHY94	-	-	++
XHY41	-	-	-	XHY95	-	+	-
XHY42	-	+	-	XHY96	-	+	-
XHY43	+	+	+	XHY97	++	-	-
XHY44	+	-	++	XHY98	+	-	+
XHY45	-	+	-	XHY99	-	-	-
XHY46	+	-	+	XHY100	+	-	++
XHY47	-	-	-	XHY101	+	-	-
XHY48	-	++	-	XHY102	-	+	-
XHY49	+	++	+	XHY103	-	+	-
XHY50	++	++	+	XHY104	++	-	-
XHY51	-	-	+	XHY105	+	-	+
XHY52	-	++	+	XHY106	-	-	-
XHY53	-	++	-	XHY107	+	-	++
XHY54	-	+	+	XHY108	+	-	-

Growth of strains is shown as follows: “++”: OD_600_ of cultures above 0.4, “+”: OD6_00_ of cultures between 0.2 and 0.4, and “-”: OD6_00_ of cultures under 0.2. The proteinase activity of strains was shown by the size of the hydrolysis circle radius, as follows: “++”: the hydrolysis circle above 5 mm, “+”: the hydrolysis circle between 1 and 5 mm, and “-”: none of the hydrolysis circle. The strains isolated from dry-cured Xuanwei ham were named starting with “XHY”.

**Table 2:** The bacteriostatic, auto-aggregation, and antioxidant activities of 27 isolates.

Strain Name	Bacteriostatic			Auto-Aggregation (%)			DPPH Scavenging Activity (%)
	*E. coli*	*S. aureus*	*Salmonella* sp.	2 h	4 h	24 h	
XHY3	-	+	-	80.49 ± 0.45 ^fgh^	86.87 ± 0.4 ^cde^	91.3 ± 0.29 ^e^	84.83 ± 0.1 ^ab^
XHY4	+	-	+	84.7 ± 0.36 ^cd^	87.94 ± 0.5 ^cd^	91.68 ± 0.07 ^d^	84.85 ± 0.08 ^ab^
XHY13	-	+	++	86.08 ± 0.19 ^abc^	90.04 ± 0.28 ^ab^	93.01 ± 0.16 ^c^	84.97 ± 0.03 ^a^
XHY15	-	-	+	88.17±0.18 ^a^	90.19 ± 0.17 ^ab^	92.89 ± 0.01 ^c^	84.46 ± 0.32 ^abc^
XHY16	+	+	+	80.81 ± 1.17 ^fg^	81.28 ± 0.05 ^j^	90.24 ± 0.01 ^fg^	84.98 ± 0.08 ^a^
XHY17	-	-	+	82.7 ± 0.29 ^def^	81.22 ± 0.69 ^j^	91.73 ± 0.05 ^d^	84.94 ± 0.08 ^a^
XHY18	-	+	-	81.37 ± 0.4 ^efg^	83.76 ± 1.05 ^ghi^	87.6 ± 0.32 ^lm^	75.54 ± 0.31 ^fghi^
XHY20	+	-	+	69.59 ± 0.06 ^j^	81.27 ± 0.39 ^j^	95.44 ± 0.04 ^a^	84.49 ± 0.14 ^bc^
XHY21	-	-	-	84.34 ± 0.71 ^cd^	86.75 ± 0.58 ^de^	89.82 ± 0.09 ^hi^	77.35 ± 0.1 ^fg^
XHY23	-	+	+	78.71 ± 1.76 ^ghi^	83.86 ± 0.41 ^ghi^	87.25 ± 0.06 ^m^	77.82 ± 0.13 ^f^
XHY27	-	+	+	71.17 ± 0.57 ^f^	74.17 ± 1.73 ^l^	82.38 ± 0.03 ^o^	79.53 ± 0.18 ^e^
XHY28	-	-	-	79.75 ± 1.04 ^gh^	83.47 ± 0.15 ^hi^	89.97 ± 0.03 ^gh^	76.87 ± 0.09 ^ghi^
XHY30	-	+	-	78.67 ± 0.08 ^ghi^	84.85 ± 0.82 ^fgh^	88.78 ± 0.11 ^k^	80.32 ± 0.38 ^d^
XHY35	+	-	++	69.93 ± 1.25 ^j^	75.67 ± 0.68 ^l^	77.62 ± 0.1 ^q^	74.45 ± 0.31 ^k^
XHY43	+	+	+	79.81 ± 0.75 ^cde^	85.51 ± 0.44 ^efg^	89.9 ± 0.06 ^gh^	76.53 ± 0.16 ^hi^
XHY44	-	-	+	83.87 ± 0.52 ^abc^	87.59 ± 0.89 ^cd^	91.76 ± 0.18 ^d^	76.5 ± 0.13 ^hi^
XHY49	+	-	+	71.43 ± 0.42 ^j^	78.77 ± 2 ^k^	79.49 ± 0.36 ^p^	76.43 ± 0.14 ^i^
XHY50	+	+	+	87.99 ± 0.02 ^a^	90.48 ± 0.65 ^a^	93.92 ± 0.03 ^b^	83.87 ± 0.03 ^c^
XHY55	+	-	+	84.94 ± 1.4 ^bcd^	88.59 ± 0.23 ^bc^	91.64 ± 0.39 ^de^	85.03 ± 0.01 ^a^
XHY69	+	+	+	87.62 ± 0.32 ^ab^	89.88 ± 0.24 ^ab^	93.85 ± 0.03 ^b^	85.08 ± 0.04 ^a^
XHY70	-	-	-	84.22 ± 0.54 ^cde^	87.83 ± 0.34 ^cd^	91.46 ± 0.37 ^de^	84.1 ± 0.2 ^abc^
XHY79	-	+	+	83.07 ± 0.39 ^def^	87.74 ± 0.23 ^cd^	90.49 ± 0.07 ^f^	84.82 ± 0.05 ^ab^
XHY83	-	-	+	77.58 ± 0.22 ^hi^	83 ± 0.4 ^i^	86.01 ± 0.08 ^n^	77.27 ± 0.20 ^fgh^
XHY88	+	+	+	83.28 ± 0.57 ^cdef^	85.64 ± 1.5 ^ef^	89.52 ± 0.08 ^ij^	77.27 ± 0.12 ^ghi^
XHY89	-	-	-	78.55 ± 2.88 ^ghi^	83.77 ± 0.8 ^fghi^	87.51 ± 0.05 ^m^	76.84 ± 0.01 ^ghi^
XHY92	-	-	+	76.71 ± 4.57 ^i^	84.21 ± 0.71 ^ghi^	87.93 ± 0.02 ^l^	77.03 ± 0.06 ^ghi^
XHY93	-	+	+	79.57 ± 1.2 ^ghi^	86.82 ± 0.2 ^cde^	89.26 ± 0.09 ^j^	76.99 ± 0.02 ^jk^

Bacteriostatic of isolates are as follows: “++”: radius of the inhibition zone above 5 mm, “+”: radius of the inhibition zone between 1 and 5 mm, and “-”: none of the inhibition zone. Values within a column with different letters are significantly different (*p* < 0.05, *n* = 4).

**Table 3:** Sequence composition and properties in the AP-D10 fraction.

ACL (%)	Sequences	Molecular Weight (Da)	Isoelectric Point (pI)	Hydrophobicity (Kcal × mol ^−1^)	Presumptive Parent Protein
99	FPPQ	487.55	5.38	7.24	Serine/threonine-protein kinase ATG1
99	VGPF	418.49	5.56	7.02	Autophagy-related protein 22
99	AGPL	356.42	5.60	8.44	Mannose-1-phosphate guanyltransferase
99	YPLP	488.58	5.48	6.22	Aminopeptidase
98	VGPV	370.44	5.63	8.27	5-methyltetrahydropteroyltriglutamate--homocysteine S-methyltransferase
98	GPFP	416.47	5.65	7.62	High-affinity K+ transporter
97	PGFP	416.47	5.25	7.62	Homoaconitase, mitochondrial
96	APGGF	447.48	5.53	9.13	Heat shock protein 70 1; vacuolar amino acid transporter 3

The properties of peptide sequences were obtained from the Pepdraw tool (http://pepdraw.com/, accessed on 15 January 2022) and the presumptive parent protein was predicted by the UniProt website (www.uniprot.org, accessed on 15 January 2022).

**Table 4:** ABTS radical scavenging activity of synthetic peptides at a concentration of 2 mg/mL.

Peptides	FPPQ	VGPF	AGPL	YPLP	VGPV	GPFP	PGFP	APGGF
ABTS scavenging activity (%)	12.83 ± 1.55 ^d^	12.78 ± 0.11 ^d^	12.79 ± 1.34 ^d^	75.48 ± 0.23 ^a^	14.76 ± 0.96 ^ce^	34.41 ± 1.34 ^b^	13.83 ± 0.15 ^d^	18.44 ± 1.47 ^c^

All values were expressed as mean ± SE (*n* = 4). Different letters (a–e) represent significant differences (*p* < 0.05).

## Data Availability

Data are contained within the article and Supplementary Material.

## Supplementary Materials

The following supporting information can be downloaded at: https://www.mdpi.com/article/10.3390/antiox11101970/s1, Table S1: The peptide content of fractions; Table S2: Sequence composition and properties in the AP-D10 fraction.

## References

[1] Wang X.H., Ma P., Jiang D.F., Peng Q., Yang H.Y. (2006). The natural microflora of Xuanwei ham and the no-mouldy ham production. J. Food Eng..

[2] Núñez F., Rodríguez M.M., Córdoba J.J., Bermúdez M.E., Asensio M.A. (1996). Yeast population during ripening of dry-cured Iberian ham. Int. J. Food Microbiol..

[3] FAO/WHO (2001). Evaluation of Health and Nutritional Properties of Powder Milk and Live Lactic Acid Bacteria.

[4] Zullo B.A., Ciafardini G. (2019). Evaluation of physiological properties of yeast strains isolated from olive oil and their in vitro probiotic trait. Food Microbiol..

[5] Hatoum R., Labrie S., Fliss I. (2012). Antimicrobial and probiotic properties of yeasts: From fundamental to novel applications. Front. Microbiol..

[6] Gil-Rodríguez A.M., Carrascosa A.V., Requena T. (2015). Yeasts in foods and beverages: In vitro characterisation of probiotic traits. LWT-Food Sci. Technol..

[7] Agarbati A., Canonico L., Marini E., Zannini E., Ciani M., Comitini F. (2020). Potential Probiotic Yeasts sourced from natural environmental and spontaneous processed foods. Foods.

[8] Klemashevich C., Wu C., Howsmon D., Alaniz R.C., Lee K., Jayaraman A. (2014). Rational identification of diet-derived postbiotics for improving intestinal microbiota function. Curr. Opin. Biotechnol..

[9] Aguilar-Toala J.E., Garcia-Varela R., Garcia H.S., Mata-Haro V., Gonzalez-Cordova A.F., Vallejo-Cordoba B., Hernandez-Mendoza A. (2018). Postbiotics: An evolving term within the functional foods field. Trends Food Sci. Technol..

[10] Canani R.B., De Filippis F., Nocerino R., Laiola M., Paparo L., Calignano A., De Caro C., Coretti L., Chiariotti L., Gilbert J.A. (2017). Specific signatures of the gut microbiota and increased levels of butyrate in children treated with fermented cow’s milk containing heat-killed *Lactobacillus paracasei* CBA L74. Appl. Environ. Microbiol..

[11] Gosalbez L., Ramon D. (2015). Probiotics in transition: Novel strategies. Trends Biotechnol..

[12] Martorell P., Alvarez B., Llopis S., Navarro V., Ortiz P., Gonzalez N., Balaguer F., Rojas A., Chenoll E., Ramon D. (2021). Heat-treated *Bifidobacterium longum* CECT-7347: A whole-cell postbiotic with antioxidant, anti-inflammatory, and gut-barrier protection properties. Antioxidants.

[13] De Almada C.N., Almada C.N., Martinez R.C.R., Sant’Ana A.S. (2016). Paraprobiotics: Evidences on their ability to modify biological responses, inactivation methods and perspectives on their application in foods. Trends Food Sci. Technol..

[14] Poprac P., Jomova K., Simunkova M., Kollar V., Rhodes C.J., Valko M. (2017). Targeting free radicals in oxidative stress-related human diseases. Trends Pharmacol. Sci..

[15] Delgado M.C.O., Nardo A., Pavlovic M., Rogniaux H., Anon M.C., Tironi V.A. (2016). Identification and characterization of antioxidant peptides obtained by gastrointestinal digestion of amaranth proteins. Food Chem..

[16] Vieira E.F., das Neves J., Vitorino R., da Silva D.D., Carmo H., Ferreira I.M.P.L.V.O. (2016). Impact of in vitro gastrointestinal digestion and transepithelial transport on antioxidant and ACE-inhibitory activities of brewer’s spent yeast autolysate. J. Agr. Food Chem..

[17] Mirzaei M., Mirdamadi S., Safavi M., Soleymanzadeh N. (2020). The stability of antioxidant and ACE-inhibitory peptides as influenced by peptide sequences. LWT-Food Sci. Technol..

[18] Mirzaei M., Shavandi A., Mirdamadi S., Soleymanzadeh N., Motahari P., Mirdamadi N., Moser M., Subra G., Alimoradi H., Goriely S. (2021). Bioactive peptides from yeast: A comparative review on production methods, bioactivity, structure-function relationship, and stability. Trends Food Sci. Technol..

[19] Zoumpopoulou G., Foligne B., Christodoulou K., Grangette C., Pot B., Tsakalidou E. (2008). *Lactobacillus fermentum* ACA-DC 179 displays probiotic potential in vitro and protects against trinitrobenzene sulfonic acid (TNBS)-induced colitis and Salmonella infection in murine models. Int. J. Food Microbiol..

[20] Xing L.J., Hu Y.Y., Hu H.Y., Ge Q.F., Zhou G.H., Zhang W.G. (2016). Purification and identification of antioxidative peptides from dry-cured Xuanwei ham. Food Chem..

[21] Ge Q., Yang B., Liu R., Jiang D., Yu H., Wu M., Zhang W. (2021). Antioxidant activity of *Lactobacillus plantarum* NJAU-01 in an animal model of aging. BMC Microbiol..

[22] Staniszewski A., Kordowska-Wiater M. (2021). Probiotic and potentially probiotic yeasts-characteristics and food application. Foods.

[23] Sambrani R., Abdolalizadeh J., Kohan L., Jafari B. (2021). Recent advances in the application of probiotic yeasts, particularly Saccharomyces, as an adjuvant therapy in the management of cancer with focus on colorectal cancer. Mol. Biol. Rep..

[24] Buzzini P., Martini A. (2002). Extracellular enzymatic activity profiles in yeast and yeast-like strains isolated from tropical environments. J. Appl. Microbiol..

[25] McFarland L.V. (2015). From yaks to yogurt: The history, development, and current use of probiotics. Clin. Infect. Dis..

[26] Ilango S., Antony U. (2021). Probiotic microorganisms from non-dairy traditional fermented foods. Trends Food Sci. Technol..

[27] Hojjati M., Behabahani B.A., Falah F. (2020). Aggregation, adherence, anti-adhesion and antagonistic activity properties relating to surface charge of probiotic *Lactobacillus brevis* gp104 against *Staphylococcus aureus*. Microb. Pathogenes..

[28] Binetti A., Carrasco M., Reinheimer J., Suarez V. (2013). Yeasts from autochthonal cheese starters: Technological and functional properties. J. Appl. Microbiol..

[29] Goktas H., Dikmen H., Demirbas F., Sagdic O., Dertli E. (2021). Characterisation of probiotic properties of yeast strains isolated from kefir samples. Int. J. Dairy Technol..

[30] Amaretti A., di Nunzio M., Pompei A., Raimondi S., Rossi M., Bordoni A. (2013). Antioxidant properties of potentially probiotic bacteria: In vitro and in vivo activities. Appl. Microbiol. Biot..

[31] Wang Y., Wu Y., Wang Y., Xu H., Mei X., Yu D., Wang Y., Li W. (2017). Antioxidant properties of probiotic bacteria. Nutrients.

[32] Gallardo G., Ruiz-Moyano S., Hernández A., Benito M.J., Córdoba M.G., Pérez-Nevado F., Martín A. (2014). Application of ISSR-PCR for rapid strain typing of *Debaryomyces hansenii* isolated from dry-cured Iberian ham. Food Microbiol..

[33] Zhang L., Huang C., Johansen P.G., Petersen M.A., Poojary M.M., Lund M.N., Jespersen L., Arneborg N. (2021). The utilisation of amino acids by *Debaryomyces hansenii* and *Yamadazyma triangularis* associated with cheese. Int. Dairy J..

[34] Mirzaei M., Mirdamadi S., Ehsani M.R., Aminlari M. (2018). Production of antioxidant and ACE-inhibitory peptides from *Kluyveromyces marxianus* protein hydrolysates: Purification and molecular docking. J. Food Drug Anal..

[35] Hsu C.N., Tain Y.L. (2019). Regulation of nitric oxide production in the developmental programming of hypertension and kidney disease. Int. J. Mol. Sci..

[36] Liu R., Xing L.J., Fu Q.Q., Zhou G.H., Zhang W.G. (2016). A review of antioxidant peptides derived from meat muscle and by-products. Antioxidants.

[37] Mirzaei M., Mirdamadi S., Ehsani M.R., Aminlari M., Hosseini E. (2015). Purification and identification of antioxidant and ACE-inhibitory peptide from *Saccharomyces cerevisiae* protein hydrolysate. J. Funct. Foods.

[38] Sarmadi B.H., Ismail A. (2010). Antioxidative peptides from food proteins: A review. Peptides.

[39] Yang W.S., Hao X.Y., Zhang X.X., Zhang G.X., Li X.D., Liu L., Sun Y., Pan Y. (2021). Identification of antioxidant peptides from cheddar cheese made with *Lactobacillus helveticus*. LWT-Food Sci. Technol..

[40] You L.J., Zhao M.M., Regenstein J.M., Ren J.Y. (2010). Purification and identification of antioxidative peptides from loach (*Misgurnus anguillicaudatus*) protein hydrolysate by consecutive chromatography and electrospray ionization-mass spectrometry. Food Res. Int..

[41] Zhang C., Zhang Y., Wang Z.Y., Chen S.W., Luo Y.K. (2017). Production and identification of antioxidant and angiotensin-converting enzyme inhibition and dipeptidyl peptidase IV inhibitory peptides from bighead carp (*Hypophthalmichthys nobilis*) muscle hydrolysate. J. Funct. Foods.

[42] Mirdamadi S., Mirzaei M., Soleymanzadeh N., Safavi M., Bakhtiari N., Zandi M. (2021). Antioxidant and cytoprotective effects of synthetic peptides identified from *Kluyveromyces marxianus* protein hydrolysate: Insight into the molecular mechanism. LWT-Food Sci. Technol..

[43] Fu Y., Young J.F., Rasmussen M.K., Dalsgaard T.K., Lametsch R., Aluko R.E., Therkildsen M. (2016). Angiotensin I-converting enzyme-inhibitory peptides from bovine collagen: Insights into inhibitory mechanism and transepithelial transport. Food Res. Int..

[44] Fu L., Xing L., Hao Y., Yang Z., Teng S., Wei L., Zhang W. (2021). The anti-inflammatory effects of dry-cured ham derived peptides in RAW264.7 macrophage cells. J. Funct. Foods.

