# Supplementary material for: Selection of Potential Probiotic Yeasts from Dry-Cured Xuanwei Ham and Identification of Yeast-Derived Antioxidant Peptides
Source: Antioxidants (Basel). 2022 Sep 30;11(10):1970. doi: 10.3390/antiox11101970 (PMC9598758; doi:10.3390/antiox11101970)
Supplement: Supplementary file 1 [file antioxidants-11-01970-s001.zip › antioxidants-1878212-supplementary.pdf]

Table S1. The peptide content of fractions.

Peptide fractions	Peptide content（%）
XHY69AP（<3KD）	97.69±1.36^{a}
XHY69AP（>3KD）	87.47±1.47^{b}
XHY79AP（<3KD）	96.56±1.20^{a}
XHY79AP（>3KD）	88.05±0.58^{b}

a-b: different lower case letter are significantly different ($P < 0.05$, n=6).

Table S2. Sequence composition and properties in the AP-D10 fraction.

No.	Confidence of sequencing (%)	Sequence	Molecular weight (kDa)	Presumptive Parent protein
1	99	FPPQ	0.488	Serine/threonine-protein kinase ATG1
2	99	VGPF	0.418	Autophagy-related protein 22
3	99	AGPL	0.356	Mannose-1-phosphate guanyltransferase
4	99	YPLP	0.489	Aminopeptidase
5	98	VGPV	0.37	5-methyltetrahydropteroyltriglutamate--homocysteine S-methyltransferase
6	98	GPFP	0.416	High-affinity K+ transporter
7	97	PGFP	0.416	Homoaconitase, mitochondrial(Q2HZ33)
8	96.1037	APGGF	0.447	Heat shock protein 70 1, Vacuolar amino acid transporter 3
9	69.2323	RICLL	0.617	Putative uncharacterized protein YFR036W-A
10	62.943	RLCIL	0.617	Putative uncharacterized protein YLR339C
11	62.5664	RLLLC	0.617	Putative uncharacterized protein YNL226W
12	58.0009	RICIL	0.617	Putative uncharacterized protein YBR013C
13	56.8785	RLCLI	0.617	SEH-associated protein 4
14	56.5698	GGLSLL	0.559	Superoxide dismutase [Mn], mitochondrial
15	54.6758	RILLC	0.617	Putative uncharacterized protein YPR177C
16	52.4139	RICLI	0.617	Probable alanine aminotransferase
17	50.2217	RLLIC	0.617	Uncharacterized protein YDR194W-A
18	47.3765	RIILC	0.617	Probable ADP-ribose 1''-phosphate phosphatase YML087W
19	46.6344	YPGNA	0.521	SMY2 homolog 2
20	41.5581	TTMPL	0.562	Putative transcription factor SEF1
21	41.2904	RILIC	0.617	DNA polymerase alpha-associated DNA helicase A
22	41.1204	DNSPQL	0.673	SPS-sensor component PTR3
23	38.5299	RIIIC	0.617	MEMO1 family protein MHO1

24	30.8236	AFVRV	0.591	Dihydrolipoyllysine-residue acetyltransferase component of pyruvate dehydrogenase complex, mitochondrial
25	30.259	LAAGPQ	0.556	Phosphoenolpyruvate carboxykinase (ATP)
26	30.0145	AGLNAG	0.502	Proteasome activator BLM10
27	29.7797	KPSPQ	0.556	Nucleoporin NUP57
28	28.0258	VGPGV	0.428	Probable diacetyl reductase [(R)-acetoin forming] 2
29	26.2233	AGINAG	0.502	Mitochondrial translation optimization protein 1
30	24.9688	ASKRR	0.617	37S ribosomal protein RSM28, mitochondrial
31	24.6725	GLASPS	0.531	Protein JSN1
32	23.6282	GIANAG	0.502	Flocculation suppression protein
33	21.3467	RAKTFT	0.723	Arginine metabolism regulation protein II
34	20.5963	PVNGA	0.457	Nicotinate-nucleotide pyrophosphorylase [carboxylating]
35	20.3688	PVAGN	0.457	SMY2 homolog 2
36	18.959	GRKRT	0.617	Peroxisome biogenesis factor 10
37	18.5455	IRTCV	0.591	Autophagy-related protein 13
38	17.9006	LMRVV	0.617	DNA repair protein RAD5
39	17.2498	GTLSTL	0.591	T-complex protein 1 subunit epsilon
40	16.7914	ATLLLS	0.617	Protoporphyrin uptake protein 1
41	15.9673	GQSSNSR	0.735	Arginine metabolism regulation protein II
42	15.2816	ATILLS	0.617	Uridine kinase
43	14.8535	AILLST	0.617	Vacuolar amino acid transporter 3
44	13.1117	ATLLIS	0.617	Putative uncharacterized protein OPI8
45	12.2059	MKELK	0.648	Dihydrolipoyllysine-residue acetyltransferase component of pyruvate dehydrogenase complex, mitochondrial
46	11.6833	ARRKS	0.617	Ubiquitin-binding protein CUE5
47	11.5538	PVSGE	0.488	Serine/threonine-protein kinase KIN2
48	11.0737	ATLIIS	0.617	Oligo-1,6-glucosidase IMA5
49	10.9064	TASLGV	0.547	26S proteasome regulatory subunit RPN2

50	10.7124	LMEKK	0.648	Sensitive to high expression protein 9, mitochondrial
51	10.1178	GTLSTI	0.591	Mitochondrial nicotinamide adenine dinucleotide transporter 1
52	9.90302	KRRAS	0.617	Flocculation suppression protein
53	5.01368	KDLSES	0.678	Nuclear fusion protein KAR5
54	4.9613	KDISES	0.678	Threonine--tRNA ligase, cytoplasmic
55	3.72299	VAEEH	0.584	Putative aryl-alcohol dehydrogenase AAD15

The properties of peptide sequences were obtained from Pepdraw tool (http://pepdraw.com/, accessed on 15 January 2022) and the presumptive parent protein was predicted by UniProt website (www.Uniprot.org, accessed on 15 January 2022).
